# Indents

**Published:** 1922-09

**Authors:** 


### INDENTS

**☞Brief Articles of Technical or Scientific Value will receive notice and comment. Contributions invited.**

---

Why My Patients Come Back. For two or three years I have used this service plan with excellent results. It is an idea which is readily adaptable to many lines of business, even hardware, for example, and provides an automatic reminder exactly when needed.

I make out a duplicate file card, containing my patient's name and postal address, at the time any particular dental work is being done. One card I keep in the regular business file; the duplicate in a separate file, indexed by the months of the year.

For instance, a patient came in for dental work in December. The duplicate card, made out following the first visit, I placed under the month index for the month of June; that is, six months ahead. Towards the end of May, all the cards that have accumulated under the index for June will be taken out and the following printed card mailed to each individual:

"Dear Mr. Blank:

"Just a friendly reminder that you owe your teeth a visit to the dentist some time next month."

While no appeal is made for the business, not one person out of a hundred receiving this brief notice would even consider visiting any other dentist. Most of the recipients regard it as a personal favor to have the matter called to their attention at the proper time. The returns from these cards, by the way, have averaged between 80% and 90%.—*System*, 1922.

---

Matrix Retainer. The ordinary rubber-dam clamp makes a very efficient and strong matrix retainer. Take a steel matrix strip, cut to proper size and contour to the tooth on which it is to be used. Remove from the mouth, and on the buccal and lingual portions of the matrix near the distal and mesial margins, punch a few perforations. The matrix is replaced on the tooth, and with clamp forceps the clamp is brought to place over the matrix so that projections on the clamp will engage the perforations on the matrix. This prevents the clamp from slipping from the matrix or springing out of place.

Do not place the clamp too far gingivally, as this will cause the matrix to buckle, but set to place just slightly below the greatest diameter of the tooth if possible; the perforations on the matrix should be made accordingly. This retainer is better than most mechanical affairs, as it is not cumbersome and does not interfere with proper manipulation and, besides, is strong and practical and does not shut off any light from the place at which the operator is working.—By Alan A. Schwimmer, in *Cosmos*.

---

Ruptured Aneurysm of the Tongue. Sabin reports a case (age thirty-four) of ruptured aneurysm of the tongue, possibly associated with pregnancy. During three previous pregnancies varicose veins of thighs had been marked; and this condition had again appeared during the fourth and present pregnancy. Venereal history negative, Wassermann not done. One week before reporting to Sabin she first noticed "a dark red blister on the tongue," pulsating synchronously with heart beat. The blister was about the size of the head of the old phosphorus match. It was located on the dorsum, in the mid line, about

2 cm. from the lip. No pain. Four days after first recognition the "blister" ruptured. Local hemostatics failed. On examination the tongue was found to be covered with a heavy black coating from the caustics. At the site of the "blister," an artery, the size of a pencil lead, was spurting blood 30-38 cm. Under local anesthesia the artery was ligated and recovery was uneventful.—By Fred C. Sabin, *Journal A. M. A.*, March, 1922.

---

INTELLIGENCE AND DECISSION IN DIAGNOSIS. A few years ago a physician called me by 'phone in regard to a patient he wanted me to treat with emetin ipecac. He said he had made slides from pockets and found amœba buccalis a plenty. He also said that this lady yielded very nicely to medicinal treatment and that we could expect a dandy result in this particular case. You all know what happened, and now we are confronted with another cure called Mercitan.

It is high time for an organized effort that will produce a condition that will make it possible for any dentist who so chooses, or desires to have the privilege of saying: We have decided so and so instead of "I think" or "I believe." "We" is so much more positive and assuring, than that hesitating, "I think." After all, progress is organization, and it advances just in proportion as men learn to act in groups; and I say this with a full knowledge of the fact that only very recently a noted educator viewed with alarm the increased tendencies of organized effort, and increase in the number of organizations, declaring that they produced a crowd-mindedness and mass-action without individual thought or effort. In the face of this however, most sociologists claim that due to too much individualism, this has become an age of immaturity of experiment, and vacillations; that it has invaded our economic life, our social life and professional life— and the dental profession has not escaped.

There is only one way in which we can become thoroughly equipped and trained to meet the vacillations and vicissitudes of the present day, and that is by an honest persistency in building up our intellectual fortification with such a definite understanding of the past, and such a careful, collective, co-operative and rational approach of the present, that we will not find ourselves either in the ranks of the ultra-radical with its biased distortions, nor in the ranks of the ultra-conservative with its deadening reaction. A real student, a real philosopher, a real practical dentist who stands square upon both feet and grows tall enough to serve all mankind, rich or poor, does not belong to either rank necessarily, as both ranks are working out the general rules and laws of progress. So I say that no idea in all the range of the human mind, in all of its excursions and ramifications is so imperative, so necessary to the eradication of the many little essences of hatred, bitterness, conflict, confusion and struggle as the idea of corporation.

Now of course, there must be individual thinking, and there is pleasure in thinking; questioning and doubt have their rewards in the mind, and can also have their liberty and exercise and reward right here within our own circle. But don't forget that the thing we call efficiency is reserved for the profession or organization that can bunch its total energy into a clear belief and hurl itself solidly at a hesitating medical profession and a hesitating world.

In conclusion, I hope that the day is not far distant that when we are counselled by our patients on questions too big for *I*, we may have the privilege, intelligence and security of saying, *WE*.—C. Olsen, in *Dental Summary* for June 1923.

---

INDIRECT INLAYS. Inlays are good in proportion to the perfection of every detail, viz.: Margins, occlusion, contact point, cavity form, which must embody form for retention, extension for prevention, and resistance to the stress of mastication.

How to best obtain a perfect inlay, providing cavity preparation has been properly made—we will assume that our cavity is prepared, giving us our resistance, proper cavo-surface angle, etc.

It hardly seems logical that with a material as frail and unresisting as inlay wax that we are able to make a perfect pattern of our cavity, trim same properly at the gingival, remove from the tooth under the difficulties we encounter in the mouth, especially in M.O.D. or D.O. cavities, and expect a very high percentage of perfection by the direct method.

The writer has been using the indirect method for some time in all posterior inlays except the very simple occlusal cavities, and the slight extra time used in making the model and articulating same is easily over-balanced by the time saved in setting the inlay when finished, as practically all the finishing can be done in the laboratory and more accurately, to a metal model which can be held in the hand, than to a tooth, in some inaccessible part of the mouth.

The impression is taken with Kerr's modeling compound cones, using a loose-fitting copper matrix which is to force the material to close contact with the tooth at the gingival, taking the impression of the greater part of the tooth. If not the whole tooth enough to carry the compound well back from the margins of the cavity. With the saliva ejector in place, chill the impression well with water, remove and see that all margins are clear and distinct, that the impression material is not distorted in withdrawal.

Next take a mash bite, using pink base plate wax, insert temporary filling and dismiss patient.

In making the amalgam model the operator has his choice of several good alloys for models, which are less expensive and more suitable than regular filling alloy. Mix more plastic than for filling, pack impression, using usual instruments for working amalgam, leave the amalgam model to set over night.

The next morning separate and place amalgam model in wax bite, pour model and articulation with good model plaster.

After model and articulation are separated, take good inlay wax, force into cavity in amalgam model, bite articulator together, remove amalgam model and carve inlay, using care to preserve the proximal contact and marginal ridge.

Carve sulci, observing the direction of stress of mastication. As a final polish we use silk; ordinary silk cloth cut into strips to polish wax.

Invest and cast in whatever manner the operator gets the best results. The discussion one hears about the shrinkage of investment sand, gold, casting at certain prescribed temperatures, and with a certain casting machine is all a matter of technic with certain operators.

The writer remembers attending a dental meeting and hearing one learned speaker talk of casting inlays in cold molds, burning the wax at certain temperatures, etc. Returned to the office and consistently spoiled the first three inlays he tried by said method.

Use the methods of casting you are accustomed to and use them consistently. If you cast with a vacuum machine, an air pressure machine, or a centrifugal machine, you will get results providing your wax pattern is absolutely correct at the start. The method in which the molten gold gets into the mold is secondary.

The item of primary importance is that the gold *does* get *into* the mold and fills every space left by the burning out of the wax pattern.

After inlay is cast, fit in amalgam model and polish, and if the technic is correct it will result in a beautiful restoration of the lost portion of the tooth.—H. Van Valkenberg, in *Summary*.

---

STERILIZATION OF SURGICAL INSTRUMENTS. The results obtained by Sanderson may be thus summarized: (1) No growth was obtained when either glass rods or scalpels on which non-spore-forming organisms were present were burned off with methyl alcohol. (2) Glass rods and scalpels could not be sterilized by burning them off with methyl alcohol when spores of B. anthracis, B. cereus, B. botulinus, B. tetani, etc., were present. The burning off of glass rods and scalpels with a methyl alcohol-formaldehyd mixture (3:1) gave excellent results except in the case of the anaërobes when grease and oil were present. When absolute sterility is desired, the burning off of a surface with an inflammable fluid is not dependable.—By E. S. Sanderson, *Journal A. M. A.*, April, 1922.

---

EPILEPSY SEQUENT TO IMPACTED MANDIBULAR THIRD MOLARS. Male, age fifteen years; epileptic crises for past two years. Family history negative. The patient came to the author's attention following an attack which began as a dull pain in the left side of the face, followed by a trembling of the jaws and a loss of consciousness for three-quarters of an hour. Radiography showed an impacted and imperfectly developed left mandibular third molar, which was extracted. A few days later a pain, similar to that on the left, was noted on the right side. Radiographically the right mandibular third molar was found extracted.

No epileptic seizures during the two months following the second extracting, the period during which the patient was under observation.—By Walter Gauley and Frantz Gauley, *Cosmos*, June, 1922.

---

ORIGIN OF THE SPIROCHETES OF THE BUCCAL AND TRACHEO-BRONCHIAL REGIONS. The spirochetes found in the mouth, in association with pyorrhea, Vincent's angina, or Castellani's disease, are almost invariably present in adults. They are not, however, to be found in infants. Apparently water or milk can not be the agency of introduction. Spirochetes are abundant in soil, particularly those soils which are moist, rich in organic matter and of a neutral or slightly alkaline reaction. Most probably they gain access to the mouth by way of particles of dirt upon fruit and vegetables.—By H. Violle, *Cosmos*, June, 1922.

---

Importance of Correct Radiograms. The understanding of the dental penetogram is something which the lay laboratory operator knows nothing of. He can learn, and he does learn quickly enough, the reading of a picture as the expression of proper or improper penetration. His experience teaches him that. His initiative and his business acumen often control his sagacity as well as his veracity. He gets away with it because neither he nor his patron has any method of checking nor keeping record of the mistakes he makes nor why he makes them. The client of the laboratory feels that he has been fairly dealt with, because he has paid for a concrete and definite mechanical operation which required a definite amount of time, and some skill, as well as apparatus with which to work. The diagnosis—that part of the picture which is the most important to have correct—he gets for nothing—a sort of gift or gratuity. Human nature the world over is the same. Where people believe or are led to believe that they can get something for nothing, or where they are getting more than a hundred cents to the dollar, they feel they are getting the better for their money, be it in the stick of candy given the child by a druggist or grocer or the extra doughnut of a baker's dozen. Barnum, of circus fame, was correct in his statement about fooling the public. They still continue to be fooled. Not so easily with circuses any more. That sport is too tame. They have gone into the gamble with their health and life at stake this time. They are willing to be fooled—they are being fooled. It is just a matter of how long they will continue to be fooled.

The public can only be fooled as long as they remain uneducated. This does not mean in this instance that the public must know all about a penetogram before it can fight against the vicious practice it is the victim of, today. It means, however, that the profession which has always been the public instructor, must draw the line more closely between that part of penetology which is purely the mechanical operation of the taking of pictures and that part of it which really vitally concerns health, life and happiness —the intelligent reading of the picture and the diagnosis of the case, the truly professional part. The difference between the mechanical part of the work which a layman can do and the highly specialized and technical part—the interpretation and diagnosis—must be clearly and definitely drawn. It is true that a trained professional man who cares to adapt himself to picture making can take better pictures than the one who shoots them only once in a while. If he does well at picture taking it may be to his advantage financially as well as in every manner to devote his entire time to such work to the exclusion of his professional duties. Such a man deserves the support of the entire profession. With him many valuable consultations may be had, and much information can be learned from him which comes as the result of his experience with the power of light penetrations of the peneto-ray, and his general knowledge of histology and pathology and the anatomy of the parts, as well as the value of the records of cases which he keeps. The placing of the confidence in a professional man by the profession teaches the public to have the same confidence. From the professional man who takes pictures at a price comparable with the lay laboratory the public can not get a diagnosis without paying for it, and most often the public pays and pays well for anything they know to be of value. Competency, therefore, in the diagnosis of the dental penetogram is the weapon of this competition. Learn to use it. The picture taking end of penetography is a mechanical bit of work, capable of being learned by a layman, the diagnosis or proper interpretation can not. Be professional men. Stand by your learning and learn more about what you should know in a professional way and let the mechanical work be done by the mechanics.—Dr. W. A. Lurie, *Dental Summary*, June, 1922.

Acuracy in Diagosis and Treatment. It is worthy of passing comment that, while we have had scathing condemnation of such procedure from various sources, at the recent joint meeting of the national organizations of the denture prosthetists and the exodontists, devoted to a consideration of desirable methods of mouth preparation, the prosthetic men were a unit in endorsing the alveolotomy operation. The only censure was directed at *over-operation*, which is conceded without question—surgical judgment, again! Consultation between the exodontist and prosthetist should precede any attempt at mouth preparation and, if possible, models should be made and studied.

If the need of the so-called surgical removal of teeth in one part of the mouth may be accentuated more than in others, it is in relation to the maxillary sinus in the maxilla and the inferior dental canal in the mandible. Novitsky as early as 1914 pointed out the apparent symptoms produced by what he called "antrum and inferior dental canal drain." These symptoms are, briefly, frontal and parietal headaches and neuralgias, eye and ear disturbances in antrum drain; and occipital headaches, "third division" neuralgia, recurrent peritonsilar abscess, trismus in the mandibular articulation, and buzzing and ringing in the ears (tinnitus arium) in the inferior dental canal disturbance.

The relation of adjacent infections to antrum disease can not be too greatly stressed, for the communication between the maxillary sinus and the ethmoid cells, sphenoidal and frontal sinuses is so intimate that an infectious process once established may involve the entire paranasal tract. The difficulty of accomplishing a cure once such infection is established should cause us to consider carefully the possibilities from either leaving periapical infectious masses *in situ* or extending infection through careless operative procedure, such as opening the antrum with forceps, forcing up tooth roots, or, in attempted curettage, plunging both curet and infected tissue into the antrum.

The potentialities of inferior dental canal drainage while not as far-reaching are equally vital. We have in both this type of case and those of antrum drain the possibilities of local as well as metastatic invasion.

Benedict has pointed out the possibility also of eye disturbances, notably iritis, originating from oral foci. The possible relation of oral infection and glaucoma while suspected is still to be definitely established.

Withal, the existence of foci of infection in and about the teeth with what evidence we have been given, can not be denied. Although we were vehement in our expressions of indignation over Hunter's arraignment of "American Dentistry" in 1910, later studies by Billings, Rosenow and many others have proven definitely that all he averred was true. Unfortunately there are many in our profession whose methods of practice have not changed one iota in these many years and whose habits of thought have kept the same pace.

In conclusion we may summarize:

1. Scientific research is vitally needed to aid in the solution of our vexing problems, and should be encouraged in every possible way.

2. As a preresuisite for definite elimination of oral infectious foci, interpretable radiograms should be demanded, and these intelligently interpreted.

3. Decision as to extraction or retention of doubtful teeth should be based upon a consideration of radiographic interpretation, symptomatology, history and clinical findings, *together with* the opinion of the medical practitioner.

4. Definitely shown periapical areas of infection call for the extraction of teeth except in most favorable cases and then only with the assumption by the patient of the burden of responsibility.

5. Extraction of teeth bearing periapical infectious masses without curettage, or without adequate currettage does not eliminate the infectious focus.

6. Such residual areas of infection are autogenetically obliterated in only an uncertain proportion of cases. Those that remain are potentially as great a source of metastatic disturbance as in their previous condition.

7. The thorough elimination of such periapical lesions assuring a complete surgical result, may be accomplished by the open-view type of extraction in cases where indicated.

8. Contrary to statements often made, no undesirable sequelae may be expected to follow the *careful, intelligent* use of this method of procedure.

Since without doubt the solution of the pulpless tooth problem will come only with the prophylactic care that will prevent decay; since that millennial period is so far in the future that we must still continue to agitate this question; and since in the absence of definite scientifically determined facts, we must base our methods of procedure upon observation, by all means let our opinions as expressed be logical, progressive and unbiased, a credit to our professional traditions—not reactionary and retrogressive.—Dr. F. F. Molt, in *Dental Summary*, for June, 1922.

---

Just Fees. I want to see my profession well compensated for their services, because I know of no work more exacting than this; and yet when I hear of some of the fees that are being demanded today, I blush lest the charge profiteering may reasonably be brought against us. And I am going to be frank enough to ask the blunt question—which is the more commendable, to insert gold fillings for $7.50 which last for thirty-eight years, or to charge $750.00 for a piece of work which is so ill-advised in construction that it goes to pieces in a year or two? I have seen the latter happen more than once. The prevalent commercialism of the age has not altogether escaped the professions in its mad career, and it would sometimes seem as if that commendable entity known as professional conscience had been administered a lethal dose.—Dr. C. N. Johnson.

